# A Mobile Self-Management App (CanSelfMan) for Children With Cancer and Their Caregivers: Usability and Compatibility Study

**DOI:** 10.2196/43867

**Published:** 2023-03-30

**Authors:** Hamed Mehdizadeh, Farkhondeh Asadi, Eslam Nazemi, Azim Mehrvar, Azade Yazdanian, Hassan Emami

**Affiliations:** 1 Health Information Technology Department School of Allied Medical Sciences Mazandaran University of Medical Sciences Sari Iran; 2 Health Information Technology and Management Department School of Allied Medical Sciences Shahid Beheshti University of Medical Sciences Tehran Iran; 3 Department of Electrical and Computer Engineering Shahid Beheshti University Tehran Iran; 4 MAHAK Pediatric Cancer Treatment and Research Center Tehran Iran

**Keywords:** Digital health, eHealth, Telehealth, mHealth, Mobile app, self-management, cancer, child, parent, caregiver, usability evaluation

## Abstract

**Background:**

Despite the increasing development of different smartphone apps in the health care domain, most of these apps lack proper evaluation. In fact, with the rapid development of smartphones and wireless communication infrastructure, many health care systems around the world are using these apps to provide health services for people without sufficient scientific efforts to design, develop, and evaluate them.

**Objective:**

The objective of this study was to evaluate the usability of CanSelfMan, a self-management app that provides access to reliable information to improve communication between health care providers and children with cancer and their parents/caregivers, facilitating remote monitoring and promoting medication adherence.

**Methods:**

We performed debugging and compatibility tests in a simulated environment to identify possible errors. Then, at the end of the 3-week period of using the app, children with cancer and their parents/caregivers filled out the User Experience Questionnaire (UEQ) to evaluate the usability of the CanSelfMan app and their level of user satisfaction.

**Results:**

During the 3 weeks of CanSelfMan use, 270 cases of symptom evaluation and 194 questions were recorded in the system by children and their parents/caregivers and answered by oncologists. After the end of the 3 weeks, 44 users completed the standard UEQ user experience questionnaire. According to the children’s evaluations, attractiveness (mean 1.956, SD 0.547) and efficiency (mean 1.934, SD 0.499) achieved the best mean results compared with novelty (mean 1.711, SD 0.481). Parents/caregivers rated efficiency at a mean of 1.880 (SD 0.316) and attractiveness at a mean of 1.853 (SD 0.331). The lowest mean score was reported for novelty (mean 1.670, SD 0.225).

**Conclusions:**

In this study, we describe the evaluation process of a self-management system to support children with cancer and their families. Based on the feedback and scores obtained from the usability evaluation, it seems that the children and their parents find CanSelfMan to be an interesting and practical idea to provide reliable and updated information on cancer and help them manage the complications of this disease.

## Introduction

Recent advances in smartphone technology have made a wide variety of apps available to the public [1] that have multiple uses in health care, such as supporting patients and providing medical services [2,3]. Since children and teenagers are active users of this technology, smartphones can be an acceptable social tool to provide education and self-management to these groups [4,5]. Studies show that the rate of learning, use, and satisfaction was high in children with technological tools such as smartphones, and this group has no problem using these tools [6]. This provides a suitable opportunity for a wide population of children and adolescents to access support, assessment, and treatment of disease-related symptoms over the internet [7-9]. Despite there being many different smartphone apps in this field, most lack proper evaluation. In fact, with the rapid development of smartphones and wireless communication infrastructure, many health systems around the world are using these apps to provide health services for people without sufficient scientific efforts to design, develop, and evaluate them [10]. A review of over 75 clinical trial studies related to mobile health showed that most of these interventions, although they were conducted in high-income countries, were of low quality, and the results were far from expected. For example, as Free and colleagues [11] reported, “Our meta-analyses show that, to date, mobile technology–based interventions for diabetes control that have statistically significant effects are small and of borderline clinical importance.”

Therefore, to facilitate an increase in effectiveness, satisfaction, and trust of users, a suitable standard method should be used to evaluate the intervention [12,13]. A usability test is one of the best ways to ensure that a product meets the users’ needs [14]. According to the International Standard Organization, usability refers to “the extent to which a product can be used by a specific user to achieve specific goals with effectiveness, efficiency, and satisfaction” [13]. Moreover, usability refers to users’ satisfaction and level of engagement with the system’s user interface, ease of use, and simplicity of learning [1] Therefore, an evaluation of usability is an important step in health interventions. Additionally, a review of the final product based on the end users’ opinions provides valuable information about the quality and usability of the product for the developer [15]. This issue is influential because usability is a key factor in the acceptance and use of any new technology in the health industry, and it can have a direct impact on users’ satisfaction. [16,17]

Thus, it seems obvious that usability testing should be a common step in the development process, even for small-scale systems [16,18]. Studies show that products with higher scores in usability evaluation tests are more desired and used, while low usability has a negative effect on user acceptance [19,20]. Without considering usability and user satisfaction, no app can expect long-term use by users. For example, most users usually spend less than 30 seconds working with a new app, and if not satisfied within that time frame, they will delete it and use other alternative apps [21]. Therefore, to ensure that the product meets user needs, its usability must be evaluated. For this purpose, we aimed to evaluate the usability of CanSelfMan, a self-management system for children with cancer and their families (which we covered in another report [22]), via the User Experience Questionnaire (UEQ) questionnaire (Multimedia Appendix 1).

## Methods

### System Description

**Figure 1:**
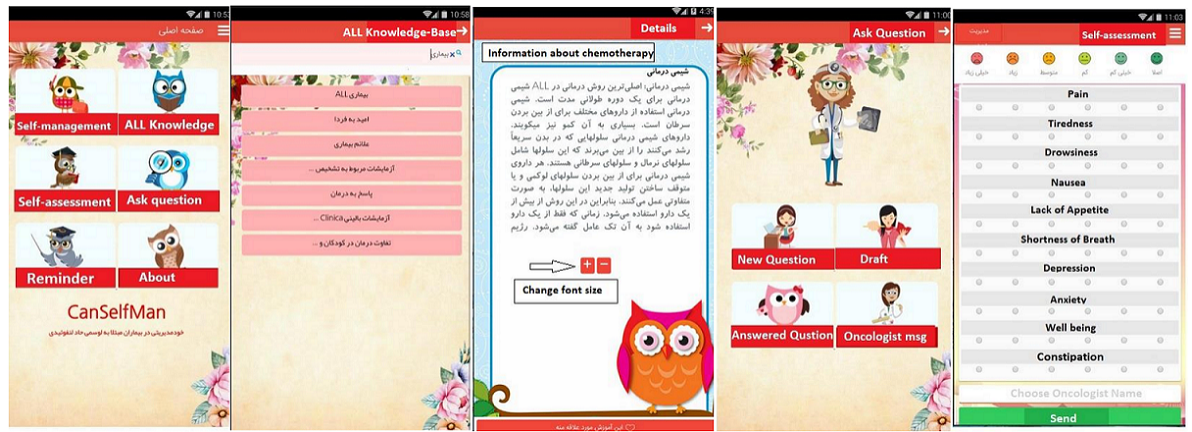
CanSelfMan app screenshots. ALL: acute lymphoblastic leukemia.

CanSelfMan is a self-management system for children with cancer that includes a web-based dashboard for oncologists and an Android app for the children and their parents/caregivers. In the initial version of CanSelfMan, there were 2 distinct versions for parents/caregivers and children. In the final iteration, a single app was made for both children and their parents/caregivers. The final version of the app had 5 modules, which included (1) cancer knowledge (ie, information about the definition of the disease, causes of the disease, treatment methods, and complications), (2) self-management recommendations (ie, recognition of symptoms, control of symptoms, physical activities, and nutritional information), (3) symptom management, (4) self-assessment questionnaire of symptoms, and (5) questions from the physician and reminders. Additionally, the oncologists’ dashboards included parts to see the results of patient assessments, questions, and answers to patients’ questions (Figure 1).

### Usability Tests

#### Performance and Compatibility Test

After we completed the steps related to coding and developing the final prototype, we performed a debugging test to evaluate app performance and identify possible errors. The process of finding and fixing errors in a software or app is called debugging, and it includes detecting codes that cause problems in app execution or performance. A debugger is a tool that helps you find and fix errors. Debugging can be done manually or through a debugger [23]. In this study, the debugging process was carried out by the principal researcher (HM) with an Android debugger.

Following this, a compatibility test was done in a simulated environment to ensure the final version of the app could run properly on different phones with different hardware and software capabilities. This test was done to check the compatibility of the app on smartphones produced by different manufacturers and with different screen sizes. For this simulation, the pCloudy [24] platform was used. This test evaluates how a mobile app performs in terms of battery consumption, memory, processor, and network data usage. It also checks how the app performs with the different smartphone brands and presents the results.

#### UEQ Evaluation

Next, to evaluate usability and user satisfaction, we used a questionnaire. After reviewing similar studies and based on the opinions of research team members, we decided to use the standard UEQ, a free questionnaire that measures usability and user experience. It has been described as a “fast and reliable questionnaire to measure the user experience of interactive products [that is] available in more than 30 languages [and is] easy to use due to rich supplementary material” [25]. It has been widely used in human-computer interaction research, and along with other qualitative evaluation methods like Think Aloud [22], it can accurately identify the weak and strong points of a product. In our previous report, we explained the results obtained from the Think Aloud evaluation [22].

The UEQ is filled out by the user after they use the product to measure its effectiveness [25]. Usually, it takes between 3 and 5 minutes to complete this questionnaire. Therefore, it is one of the most efficient methods to measure users’ opinions about a software product. The official version of this questionnaire has been translated into over 20 different languages, including Persian. It has 26 questions that include 6 measures, namely, (1) attractiveness, (2) perspicuity, (3) efficiency, (4) dependability, (5) stimulation, and (6) novelty in the 2 axes of design quality and use quality. Additionally, it measures both usability and user satisfaction with the product [26]. Textbox 1 briefly explains each of these 6 measures.

The remaining 5 scales have an impact on the attractiveness scale, which measures the user’s overall impression of the app. While stimulation and novelty describe hedonic (non–goal-directed) quality criteria, the scales of perspicuity, efficiency, and dependability provide information about pragmatic (goal-directed) quality aspects. Each item is a pair of opposites with a 7-point Likert scale, which is the form of a semantic differential. [27]. Multimedia Appendix 1 shows the UEQ items.

The UEQ has a tool designed in Microsoft Excel (Microsoft Corp) to calculate and interpret the results, as well as a database containing the results of previous studies, allowing us to compare our results with those of 246 previous studies [25,28]. The answer to each question is on a 7-point Likert scale ranging from −3 (completely agree with the negative conditions) to +3 (completely agree with the positive opinion), with 0 being neutral. If the total score of each measure is less than −0.8, it means unacceptable or weak, −0.8 to +0.8 means acceptable, and +0.8 to 3 is considered good and excellent.

**The 6 measures of the User Experience Questionnaire (UEQ).**

1. Attractiveness: Do users like or dislike the app?
2. Perspicuity: Is it easy to get used to and understand how to use the app?
3. Efficiency: Can users get their work done quickly and efficiently?
4. Dependability: Are interactions with the app safe and predictable?
5. Stimulation: Is using the app enjoyable and motivating?
6. Novelty: Do users feel motivated to continue using the app?

### Ethics Approval

This study received ethical review approval from Shahid Beheshti University of Medical Sciences (IR.SBMU.RETECH.REC.1396.1316).

### Study Setting and Population

After compatibility and technical testing, the final version was provided to end users to evaluate usability. For this purpose, a banner was placed in the outpatient department of MAHAK’s Pediatric Cancer Treatment and Research Center inviting people to participate in the study. The inclusion criteria for entering this study for children were (1) children with acute lymphoid leukemia referred to MAHAK who were in the phase of chemotherapy treatment (ie, at least 1 year had passed since the start of treatment), (2) at least 7 years of age, and (3) can work with a smartphone. For parents/caregivers, the inclusion criteria were (1) having at least 1 child with cancer that has been diagnosed for a year and is currently undergoing active treatment with chemotherapy, (2) having the ability to read and write in Persian, and (3) having an Android smartphone (version 8 to 11). The exclusion criteria were patients who were in the end stages of cancer and those struggling with their mental health and parents/caregivers who did not know how to work a smartphone and could not read or write. In addition, 4 oncologists participated in this study to answer patients’ questions.

Before initiating the study, we presented information about the study to the participants in a 30-minute session. In this session, the principal researcher (author HM) explained the app to the participants and the purpose of this study in plain language. Following that, another researcher (author AM) obtained permission from the children’s parents/caregivers to participate in this study and then from the children themselves. After this, the parents signed the consent form.

We explained to all participants that they could withdraw at any stage of the study without providing a reason. After this, the CanSelfMan app was installed on their smartphones, and they were shown how to use the app. After 3 weeks of use, the participants returned to MAHAK in person and completed a questionnaire related to demographic information including age, sex, place of residence, and education, along with the UEQ.

## Results

### CanSelfMan Compatibility Test

**Figure 2:**
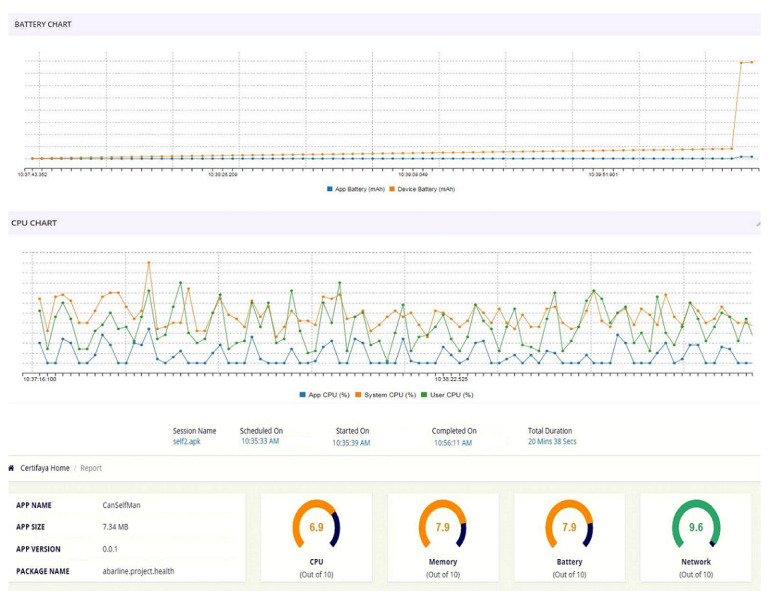
CanSelfMan app results for the compatibility test. CPU: Central Processing Unit; mAh: milliampere hour.

At this stage, to ensure that the final app was functioning correctly, a compatibility test was performed using a simulation with the pCloudy service. The results are shown in Figure 2.

One of the important issues in the compatibility test included installing, running, and uninstalling the app, which was done on 10 different brands of smartphones in the pCloudy platform.

The app was installed and run on all the desired phones without any errors. Then, the app was examined from compatibility and applicability aspects on different smartphones with different screen sizes. Again, it was executed without any problems. Next, 4 features, namely, memory, processor, network data, and battery consumption, were examined. Based on the results of this simulation, CanSelfMan acquire scored 7.9 out of 10 possible points in the battery consumption section, which is considered a good score and indicates an optimal consumption of battery and memory. Moreover, in the network exchange data evaluation, it obtained a score of 9.6, which is a very high score (Figure 2).

### Usability and User Satisfaction

During the 3 weeks of app use, 270 symptom evaluations and 194 questions were recorded in the system by children and their parents/caregivers and answered by oncologists. After the end of the 3-week period, 44 users completed the UEQ. Respondents included 25 parents who had a child under the age of 7 years with cancer.

The age range of the parents/caregivers in this phase was between 27 and 48 years, with an average age of 32 years, and most (n=16, 64%) were female. Table 1 shows the participants’ demographic information.

A total of 19 children with cancer ranging in age from 7 to 14 years, with an average age of 12 years, completed the UEQ. The majority (11/19, 58%) of the children were female. The range of points that can be obtained in UEQ is from −3 (the lowest possible point) to +3 (the maximum point that can be obtained). The results are interpreted based on average values, and there is no unique score. In addition, the CI of the measurements refers to the level of accuracy in estimating the average. As a result, the smaller the CI, the higher accuracy and reliability of the obtained results. Since each of the 6 measures in UEQ includes a set of items, none of the items can be interpreted alone. Therefore, for each measure, the total score of the related items was calculated.

**Table 1:** Participants’ demographic information.

Participants		Parents (n=25)	Children (n=19)
Age (years), mean (SD; range)		32 (2.1; 27-48)	10 (1.7; 7-14)
**Gender, n (%)**			
	Female	16 64))	11 (58)
	Male	9 (36)	8 (42)
**Residence, n (%)**			
	Urban	14 (56)	13(68)
	Rural	11 (44)	6(32)
**Education level, n (%)**			
	Secondary school	1 (4)	9 (47)
	College diploma	13 (52)	N/A^a^
	Junior college	3 (12)	N/A
	Bachelor’s degree and above	8 (32)	N/A

^a^N/A: not applicable.

The results showed the measures of transparency, motivation, and attractiveness had the highest possible points, respectively. Table 2 shows the average score and SD of each measure for both groups (ie, parents/caregivers and children). According to the children’s evaluations, attractiveness (mean 1.956, SD 0.547) and efficiency (mean 1.934, SD 0.499) achieved the best results compared with novelty (mean 1.711, SD 0.481). Parents/caregivers rated efficiency at a mean of 1.880 (SD 0.316) and attractiveness at a mean of 1.853 (SD 0.331). The lowest score was reported for novelty (mean 1.670, SD 0.225). In addition, to determine the app quality, the UEQ shows the overall performance of the product with 3 measures (attractiveness, quality of use, and quality of design) [25].

UEQ parameters can also be divided into 2 general groups: pragmatic quality (perspicuity, efficiency, dependability) and hedonic quality (stimulation and originality). Table 3 shows the app’s pragmatic and hedonic quality scores. Pragmatic quality describes task-related quality aspects, and hedonic quality describes non–task-related quality aspects [25].

According to the obtained results, CanSelfMan scored above +0.8 in all measures, which is above the average score of 1.5, thus falling in the good category. The results of the evaluation showed that the CanSelfMan app ranked highest in attractiveness and efficiency and lowest in novelty, which indicates a high level of user satisfaction with the app’s quality and user interface. One of the reasons for the app's attractive user interface could be the use of graphic elements and gamification, which attracted children’s attention and subsequently increased scores in the efficiency and app usage scales. In addition, the low score on the novelty scale may be because new apps do not evoke a sense of innovation and novelty for young users due to the increasing number of apps that are designed for this age group.

For the parents/caregivers, the app scored highly for efficiency and attractiveness and lowest for novelty. Obtaining a high score in efficiency indicates the app's quality and the high satisfaction of users regarding the use of the app. One of these reasons for this can be the integrated and modular design of the app, which, by separating the functions of each part and reducing the complexity of the app as much as possible, has made it easy for users to perform tasks and use the app. Accordingly, a high score in the attractiveness category for parents can also be a sign of the quality of the app’s user interface design, which led to high user satisfaction. The low score for novelty could be attributed to the increase in health apps in this domain available through app stores; thus, a new app like CanSelfMan may not excite or delight users enough.

**Figure 3:**
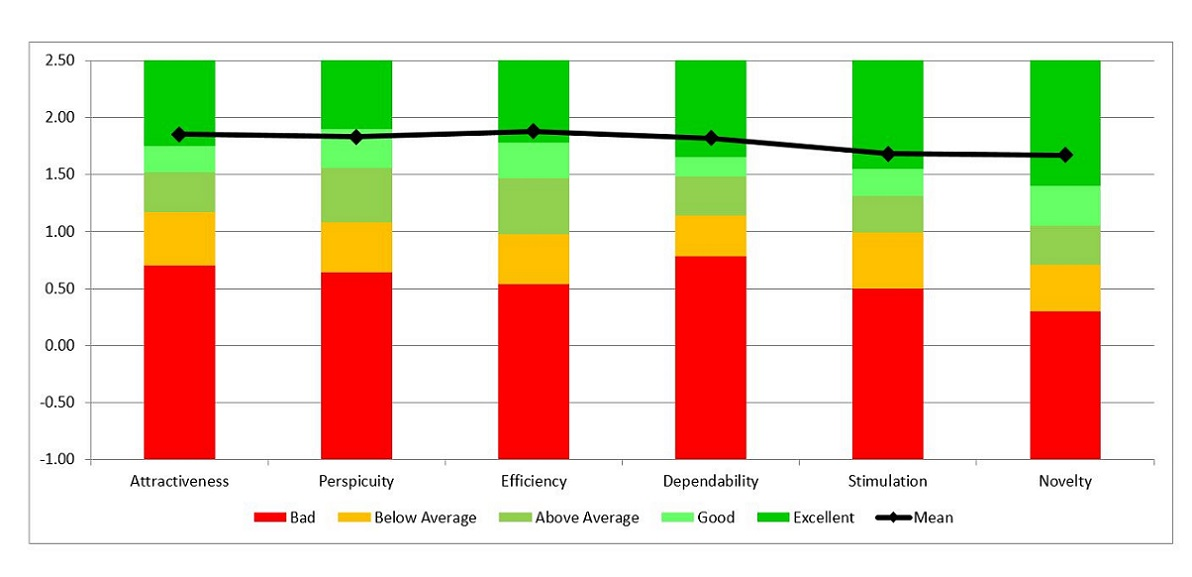
Comparison chart of the CanSelfMan app's usability results and those of previous studies.

The UEQ also provides a calculation tool that includes evaluation data from previous studies. Therefore, the results obtained in each evaluation can be compared with previous studies. To provide a better overall picture of the app quality, the results were compared with the benchmark data set of the UEQ. The data set was collected for 246 studies evaluating various products, including, web pages, mobile apps, social networks, and so forth. A comparison of the results of this study with those of previous studies indicates the relative quality of CanSelfMan on an international scale. Figure 3 shows the CanSelfMan results compared to those of 246 previous studies conducted with the UEQ.

**Table 2:** Results of the User Experience Questionnaire (UEQ) for both study groups (parents/caregivers and children).

Measures	Children (n=19)			Parents/caregivers (n=25)		
Scales	Mean (SD)	95% CI		Mean (SD)	95% CI	
Attractiveness	1.956 (0.547)	1.710-2.202		1.853 (0.481)	1.724-1.983	
Perspicuity	1.895 (0.668)	1.594-2.195		1.830 (0.481)	1.678-1.982	
Efficiency	1.934 (0.668)	1.710-2.159		1.880 (0.316)	1.756-2.004	
Dependability	1.803 (0.668)	1.567-2.038		1.820 (0.255)	1.720-1.920	
Stimulation	1.776 (0.399)	1.597-1.956		1.680 (0.223)	1.593-1.767	
Novelty	1.711 (0.481)	1.494-1.927		1.670 (0.225)	1.582-1.758	

**Table 3:** The CanSelfMan app’s pragmatic and hedonic quality scores.

Study groups	Children	Parents/caregivers
Attractiveness	1.96	1.85
Pragmatic quality	1.88	1.84
Hedonic quality	1.74	1.68

## Discussion

### Principal Findings

Information systems researchers [1] have confirmed the importance of evaluating product usability. In this study, we evaluated the usability of CanSelfMan from the aspects of technical performance and usability. CanSelfMan is an educational self-management app aimed at supporting children with cancer and their parents/caregivers, which provides access to up-to-date and reliable information about cancer and information on how to deal with and manage symptoms related to cancer.

In the first step, the technical performance of the app was examined. In general, issues such as screen size, screen resolution, processing ability, and the amount of system resource usage are considered common problems and limitations of smartphones [13]. These limitations, especially frequent outages in communication or connection to the network, variable bandwidth, and high-energy consumption can have negative effects on app quality, especially on usability and reliability [29]. Various studies have been conducted that focus on the challenges related to the usability of these tools, including the ability to run on devices with different screen sizes and the consumption of system resources including the processor, battery, system memory, and connection speed [30]. The results of these studies show that well-designed apps increase app usage and user satisfaction [31]. On the other hand, considering that different phone manufacturers make smartphones with different technical features [32], ensuring the optimality of the apps and their ability to run on and adapt to a wide range of devices with different technical characteristics is essential. Therefore, we tried to ensure the optimality of CanSelfMan via a simulation, thereby reducing the negative effects on the usability of the final version.

Since one of the limitations of smartphones is the limited amount of battery or charge maintenance, one of the most important issues relating to apps is the optimal use of this limited energy [32]. The results of the simulation and the score of 7.9 out of 10 possible points for battery consumption in different devices indicated an acceptable overall score. These features indicated that the app operated correctly, with the absence of additional load in terms of energy consumption and overall device memory usage. Moreover, this simulation provided information about the optimal execution of the app on devices with different screen sizes and showed that it was compatible with screens of different sizes and ran without any issues.

After the performance evaluation, we evaluated the CanSelfMan app from the usability aspect. Usability measurement studies can facilitate a better understanding of user interactions with the final product and help determine the strengths and weaknesses of the product according to different user groups. The results obtained from these evaluations can provide important information about user behaviors and tendencies when it comes to new technologies, thereby providing a better understanding of user acceptance for the developer [33]. As such, app developers, especially those in the health care domain, should focus on users’ needs and ensure the practicality and effectiveness of the product [34]. To this end, it is essential to perform usability tests to ensure that the final product meets the users’ needs [35]. Accordingly, we used the UEQ to evaluate the app's usability.

The results of the UEQ provided useful information about various aspects of the app. We were also able to compare various aspects of our app relating to design and performance with other similar software. This questionnaire has been widely used in human-computer interaction studies and is considered an efficient and accurate method of measuring users’ feelings toward software products. The validity and reliability of this questionnaire are very high, and it helped us obtain a comprehensive evaluation of the feelings and experiences of the users toward the CanSelfMan app. Similarly, Salari and colleagues [36] used this questionnaire to evaluate an educational and self-management app designed to support people with type 2 diabetes.

Subsequently, the final evaluation of the CanSelfMan was carried out by the children with cancer and their parents/caregivers. For both groups, attractiveness, motivation, and efficiency scales scored higher than others, indicating their satisfaction with the user interface and graphic elements used in the app, which could motivate them further to use the app. Additionally, the high scores on the efficiency, transparency, and reliability scales indicate the app’s quality and high user satisfaction. Similarly, MacPherson and colleagues [37] designed a mobile app called C-SCAT to measure and report symptoms related to chemotherapy in children and adolescents with cancer. It also provided information about symptoms, possible causes, mitigating or aggravating factors, and self-management tips to control symptoms. The results related to the usability evaluation indicated the ease of use, applicability, and high satisfaction of users of the C-SCAT app.

In another study, Wang and colleagues [38] developed a mobile app to collect, record, and assess symptoms in children with cancer and their families. The user interface of this app was designed appropriately for children, and animation and attractive colors were used. The results of the evaluation showed that children and parents felt that the app was easy to use.

However, this may raise the question of whether children have the competence and ability to evaluate app usability. The answer is yes. Currently, there are many apps designed specifically for children, and due to the increasing use of tablets and smartphones, children have a high ability to use these tools [39,40]. Moreover, based on the studies conducted in this field, children between the ages of 6 and 10 years can understand and follow instructions—skills that they learn in school. Therefore, they can complete the evaluation without any challenges. This also extends to children aged 11 to 14 years, who also have no issue in this regard due to their familiarity with computers and digital devices. [41,42]. Moreover, having children evaluate usability can be a valuable problem-solving process for app designers and help them understand how children use the product. In a study similar to ours, Massoud and colleagues [39] evaluated the usability and user interface of an educational app aimed at 4- to 5-year-old preschool children. In another study, Brown and colleagues [43] evaluated the usability of an educational app about nutrition and diet called Foodbot Factory. In their study, children aged between 9 and 12 years evaluated the app’s usability, and the results showed that the majority of them found the app easy to use and fun.

In another study, Grasaas and colleagues [6] investigated the usability of the iCanCope app, which was designed to teach adolescents with cancer self-management and pain control. It was evaluated via the System Usability Scale, with the results showing that iCanCope scored high in usability and user satisfaction. Cheng and his colleagues [44] evaluated a mobile app aimed at supporting families and children with complex conditions and family-delivered enteral tube care. In this study, the children and their families completed a questionnaire to evaluate the app’s usability. The level of user satisfaction and app usability was high, and users declared that they would recommend this app to others. In our study, both groups of users (children and their parents/caregivers) were generally satisfied with the quality of CanSelfMan.

### Weaknesses, Strengths, and Limitations

This study had several limitations. These include the small sample size and the low diversity of participants (all participants were from the same treatment center) in the evaluation stages, which makes it impossible to generalize the results of this study. However, small sample sizes in early evaluation studies usually provide adequate information about implementations. Another limitation of this study was that the usability test was conducted with the participation of elementary school children. To create a sense of confidence and facilitate cooperation, the children were accompanied by 1 parent. Additionally, while the children were completing the UEQ, one of the researchers (authors HM or AM) was present with them and explained all the options to them so that they could answer the questions. Finally, although this study was able to collect useful data on the app usability and user satisfaction through the questionnaire, the next step for future research could be to assess additional indicators such as the level of digital health literacy, the level of access to technology, and the clinical outcomes related to using this app.

### Conclusions

In this study, we described the evaluation process of CanSelfMan, a self-management app designed to support children with cancer and their families. We adopted a user-centered strategy and involved end users at every stage of app development and evaluation to ensure it was in line with the users’ requirements. To this end, the usability evaluation was carried out with the aim of solving potential issues with the app to develop a final product that would be user friendly and acceptable. The results of this study show that we were successful in achieving this goal.

Based on the feedback and the scores obtained from the usability evaluation, the children and their parents/caregivers found CanSelfMan to be an appealing and practical tool to provide reliable, up-to-update information on cancer and help them manage the complications of this health condition. However, because our small sample size prevents the generalization of our study results, other studies are needed to evaluate the usability of this app with a larger population. In future studies, we plan to investigate the clinical outcomes and the effects of the short versus long-term use of this app.

## Appendix

Multimedia Appendix 1 User Experience Questionnaire (UEQ) items.

## Abbreviations

UEQ: User Experience Questionnaire

## References

[1] Kasali FA, Awodele O, Kuyoro S, Akinsanya A, Eze M (2017). A conceptual design and evaluation framework for mobile persuasive health technologies (usability approach). Res J Math Comput Sci.

[2] Annah A, Anna M, Lori W (2016). Pediatric Psychosocial Oncology: Textbook for Multidisciplinary Care.

[3] Wesley K, Fizur P (2015). A review of mobile applications to help adolescent and young adult cancer patients. Adolesc Health Med Ther.

[4] de la Vega R, Roset R, Castarlenas E, Sánchez-Rodríguez E, Solé E, Miró J (2014). Development and testing of painometer: a smartphone app to assess pain intensity. J Pain.

[5] Stinson J, McGrath P, Hodnett E, Feldman B, Duffy C, Huber A, Tucker L, Hetherington R, Tse S, Spiegel L, Campillo S, Gill N, White M (2010). Usability testing of an online self-management program for adolescents with juvenile idiopathic arthritis. J Med Internet Res.

[6] Grasaas E, Fegran L, Helseth S, Stinson J, Martinez S, Lalloo C, Haraldstad K (2019). iCanCope With Pain: cultural adaptation and usability testing of a self-management app for adolescents with persistent pain in Norway. JMIR Res Protoc.

[7] Slater H, Stinson JN, Jordan JE, Chua J, Low B, Lalloo C, Pham Q, Cafazzo JA, Briggs AM (2020). Evaluation of digital technologies tailored to support young people's self-management of musculoskeletal pain: mixed methods study. J Med Internet Res.

[8] Stinson J, Wilson R, Gill N, Yamada J, Holt J (2009). A systematic review of internet-based self-management interventions for youth with health conditions. J Pediatr Psychol.

[9] Mehdizadeh H, Asadi F, Mehrvar A, Nazemi E, Emami H (2019). Smartphone apps to help children and adolescents with cancer and their families: a scoping review. Acta Oncologica.

[10] Ben-Zeev D, Kaiser S, Brenner C, Begale M, Duffecy J, Mohr D (2013). Development and usability testing of FOCUS: a smartphone system for self-management of schizophrenia. Psychiatr Rehabil J.

[11] Free C, Phillips G, Galli L, Watson L, Felix L, Edwards P, Patel V, Haines A (2013). The effectiveness of mobile-health technology-based health behaviour change or disease management interventions for health care consumers: a systematic review. PLoS Med.

[12] Jake-Schoffman D, Silfee V, Waring M, Boudreaux E, Sadasivam R, Mullen S, Carey JL, Hayes RB, Ding EY, Bennett GG, Pagoto SL (2017). Methods for evaluating the content, usability, and efficacy of commercial mobile health apps. JMIR Mhealth Uhealth.

[13] Harrison R, Flood D, Duce D (2013). Usability of mobile applications: literature review and rationale for a new usability model. J Interact Sci.

[14] Earthy J, Jones B, Bevan N, Buie E, Murray D (2012). ISO standards for user-centered design: the specification of usability. Usability in Government Systems: User Experience Design for Citizens and Public Servants.

[15] Stinson J, Gupta A, Dupuis F, Dick B, Laverdière C, LeMay S, Sung L, Dettmer E, Gomer S, Lober J, Chan CY (2015). Usability testing of an online self-management program for adolescents with cancer. J Pediatr Oncol Nurs.

[16] Demiris G, Afrin LB, Speedie S, Courtney KL, Sondhi M, Vimarlund V, Lovis C, Goossen W, Lynch C (2008). Patient-centered applications: use of information technology to promote disease management and wellness. A white paper by the AMIA Knowledge in Motion Working Group. J Am Med Inform Assoc.

[17] Junnu P (2016). Mobile Application Usability Research: Case Study of a Video Recording and Annotation Application. Thesis.

[18] Mourouzis A, Chouvarda I, Maglaveras N (2015). mHealth: common usability and user experience practices and flaws.

[19] Hyzy M, Bond R, Mulvenna M, Bai L, Dix A, Leigh S, Hunt S (2022). System Usability Scale benchmarking for digital health apps: meta-analysis. JMIR Mhealth Uhealth.

[20] van der Velde M, Valkenet K, Geleijn E, Kruisselbrink M, Marsman M, Janssen LM, Ruurda JP, van der Peet DL, Aarden JJ, Veenhof C, van der Leeden M (2021). Usability and preliminary effectiveness of a preoperative mHealth app for people undergoing major surgery: pilot randomized controlled trial. JMIR Mhealth Uhealth.

[21] Liew MS, Zhang J, See J, Ong YL (2019). Usability challenges for health and wellness mobile apps: mixed-methods study among mHealth experts and consumers. JMIR Mhealth Uhealth.

[22] Mehdizadeh H, Asadi F, Emami H, Mehrvar A, Nazemi E (2022). An mHealth sellf-management system for support children with acute lymphocytic leukemia and their caregivers: qualitative co-design study. JMIR Form Res.

[23] (2020). Debuggers: important tools for troubleshooting in software. Digital Guide IONOS.

[24] pCloudy.

[25] User Experience Questionnaire.

[26] Schrepp M, Hinderks A, Thomaschewski J (2017). Construction of a benchmark for the User Experience Questionnaire (UEQ). Int J Interact Multimed Artif Intell.

[27] Truong MT, Nwosu OB, Gaytan Torres ME, Segura Vargas MP, Seifer A, Nitschke M, Ibrahim AA, Knitza J, Krusche M, Eskofier BM, Schett G, Morf H (2022). A yoga exercise app designed for patients with axial spondylarthritis: development and user experience study. JMIR Form Res.

[28] Schrepp M, Hinderks A (2011). Design, user experience, and usability. Theory, methods, tools and practice. http://link.springer.com/10.1007/978-3-642-21708-1.

[29] Moumane K, Idri A, Abran A (2016). Usability evaluation of mobile applications using ISO 9241 and ISO 25062 standards. Springerplus.

[30] Ismail N, Ahmad F, Kamaruddin NI (2016). A review on usability issues in mobile applications. J Mob Comput Appl.

[31] Hoehle H, Aljafari R, Venkatesh V (2016). Leveraging Microsoft׳s mobile usability guidelines: conceptualizing and developing scales for mobile application usability. Int J Hum Comput Stud.

[32] Idri A, Moumane K, Abran A (2013). On the use of software quality standard ISO/IEC9126 in mobile environments.

[33] Bird M, Carter N, Lim A, Kazmie N, Fajardo C, Reaume S, McGillion MH (2022). A novel hospital-to-home system for children with medical complexities: usability testing study. JMIR Form Res.

[34] Johnson SG, Potrebny T, Larun L, Ciliska D, Olsen NR (2022). Usability methods and attributes reported in usability studies of mobile apps for health care education: scoping review. JMIR Med Educ.

[35] Paz F, Pow-Sang JA (2016). A systematic mapping review of usability evaluation methods for software development process. Int J Softw Eng its Appl.

[36] Salari R, Niakan Kalhori SR, GhaziSaeedi M, Jeddi M, Nazari M, Fatehi F (2021). Mobile-based and cloud-based system for self-management of people with type 2 diabetes: development and usability evaluation. J Med Internet Res.

[37] Macpherson C, Linder L, Ameringer S, Erickson J, Stegenga K, Woods N (2014). Feasibility and acceptability of an iPad application to explore symptom clusters in adolescents and young adults with cancer. Pediatr Blood Cancer.

[38] Wang J, Yao N, Liu Y, Geng Z, Wang Y, Shen N, Yuan C (2017). Development of a smartphone application to monitor pediatric patient-reported outcomes. Comput Inform Nurs.

[39] Masood M, Thigambaram M (2015). The usability of mobile applications for pre-schoolers.

[40] Mohd Azam O, Abdullah Zawawi T, Zainal Abidin S, Tan S-Y, Abdullah Sani A (2012). A study of the trend of smartphone and its usage behavior in Malaysia. Int J New Comput Archit Appl.

[41] Hanna L, Risden K, Alexander K (1997). Guidelines for usability testing with children. Interactions.

[42] Libby H, Neapolitan D, Risden K (2004). Evaluating computer game concepts with children.

[43] Brown JM, Savaglio R, Watson G, Kaplansky A, LeSage A, Hughes J, Kapralos B, Arcand J (2020). Optimizing child nutrition education with the Foodbot Factory mobile health app: formative evaluation and analysis. JMIR Form Res.

[44] Cheng C, Werner N, Doutcheva N, Warner G, Barton HJ, Kelly MM, Ehlenbach ML, Wagner T, Finesilver S, Katz BJ, Nacht C, Coller RJ (2020). Codesign and usability testing of a mobile application to support family-delivered enteral tube care. Hosp Pediatr.

