# Supplementary material for: A Mobile Self-Management App (CanSelfMan) for Children With Cancer and Their Caregivers: Usability and Compatibility Study
Source: JMIR Pediatr Parent. 2023 Mar 30;6:e43867. doi: 10.2196/43867 (PMC10132021; doi:10.2196/43867)
Supplement: Multimedia Appendix 1 [file pediatrics_v6i1e43867_app1.docx]

|  | 1 | 2 | 3 | 4 | 5 | 6 | 7 |  | **Attractiveness** =  The product should be visually appealing, entertaining, approachable, and pleasant. |
| --- | --- | --- | --- | --- | --- | --- | --- | --- | --- |
| Good |  |  |  |  |  |  |  | Bad |  |
| Unlikable |  |  |  |  |  |  |  | Pleasing |  |
| Unpleasant |  |  |  |  |  |  |  | Pleasant |  |
| Friendly |  |  |  |  |  |  |  | Unfriendly |  |
| Attractive |  |  |  |  |  |  |  | Unattractive |  |
| Annoying |  |  |  |  |  |  |  | Enjoyable |  |
| Creative |  |  |  |  |  |  |  | Dull | **Novelty** =  The product should be innovative, original, and creatively designed. |
| Inventive |  |  |  |  |  |  |  | Conventional |  |
| Usual |  |  |  |  |  |  |  | Leading edge |  |
| Conservative |  |  |  |  |  |  |  | Innovative |  |
| Fast |  |  |  |  |  |  |  | Slow | **Efficiency** =  Product tasks must be completed quickly, efficiently and practically. |
| Inefficient |  |  |  |  |  |  |  | Efficient |  |
| Impractical |  |  |  |  |  |  |  | Practical |  |
| Organized |  |  |  |  |  |  |  | Cluttered |  |
| Valuable |  |  |  |  |  |  |  | Inferior | **Stimulation** =  It should be exciting, inspiring, and fascinating to use the product. |
| Boring |  |  |  |  |  |  |  | Exciting |  |
| Not Interesting |  |  |  |  |  |  |  | Interesting |  |
| Motivating |  |  |  |  |  |  |  | Demotivating |  |
| Not Understandable |  |  |  |  |  |  |  | Understandable | **Perspicuity** =  The product should be easy to learn. |
| Easy to Learn |  |  |  |  |  |  |  | Difficult to Learn |  |
| Clear |  |  |  |  |  |  |  | Confusing |  |
| Complicated |  |  |  |  |  |  |  | Easy |  |
| Unpredictable |  |  |  |  |  |  |  | Predictable | **Dependability**=  The product engagement should be predictable, secure, and satisfy my expectations. |
| Obstructive |  |  |  |  |  |  |  | Supportive |  |
| Secure |  |  |  |  |  |  |  | Not Secure |  |
| Meets Expectations |  |  |  |  |  |  |  | Does not meet Expectations |  |

**User Experience Questionnaire (UEQ) items.**
